# Clinical study of the preventive effect of pulsed radiofrequency and continuous epidural block on postherpetic neuralgia: a randomized controlled trial

**DOI:** 10.3389/fpain.2026.1784995

**Published:** 2026-05-26

**Authors:** Ping Xu, Yong Fei, Enming Zhang

**Affiliations:** 1 Department of Anesthesiology and Pain, The Affiliated Hospital of Jiaxing University, Jiaxing, China; 2 Department of Anesthesiology, The Second Affiliated Hospital of Jiaxing University, Jiaxing, China

**Keywords:** continuous epidural block, herpes zoster, postherpetic neuralgia, pulse radiofrequency, visual analogue scale

## Abstract

**Objective:**

In this study, patients with acute herpes zoster neuralgia were respectively treated with continuous epidural block and pulsed radio frequency therapy to compare the similarities and differences of the two treatment methods in preventing the incidence of PHN, as well as the efficacy of acute herpes zoster neuralgia and observe the surgical complications.

**Methods:**

Ninety patients with herpes zoster neuralgia whose course of disease was less than 1 month were randomly divided into two groups according to the type of surgery: Continuous Epidural Block (CEB group) and Pulsed Radio Frequency (PRF group). The incidence of PHN in January, March and June was recorded. VAS scores, PSQI scores and the dosage of gabapentine capsules and Tramadol hydrochloride sustained-release tablets were recorded before surgery and 1 day, 1 week, 1 month, 3 months and 6 months after surgery. Peripheral blood Gal-3 and IL-6 levels before surgery, 1 week, 2 weeks and 4 weeks after surgery; Surgical complications; The duration of hospital stay and so on were used to evaluate the clinical effect of the operation.

**Results:**

The incidence of PHN in CEB group at 1 month, 3 months and 6 months after surgery was lower than that in PRF group, and the differences were statistically significant (*P* < 0.05). Pain intensity and PSQI were decreased in all patients after treatment. Compared with PRF group, VAS score and PSQI in CEB group decreased at 1 week, 1 month, 3 months and 6 months after surgery, and the differences were statistically significant (*P* < 0.05). Compared with before treatment, the levels of Gal-3 and IL-6 in peripheral blood of all patients were decreased after treatment, and the differences were statistically significant (*P* < 0.05). Compared with PRF group, peripheral blood Gal-3 and IL-6 levels in CEB group decreased at 1 and 4 weeks after treatment, and the differences were statistically significant (*P* < 0.05). The doses of gabapentin capsules and Tramadol hydrochloride sustained-release tablets in CEB group were lower than those in PRF group at 1 month, 3 months and 6 months after surgery, and the differences between the two groups were statistically significant (*P* < 0.05). There was one patient with puncture site infection in the CEB group, and the hospital stay in the CEB group was longer than that in the PRF group.

**Conclusions:**

Both methods can significantly relieve the pain of patients with acute herpes zoster neuralgia and reduce the incidence of PHN, but there is a risk of puncture site infection., and long hospital stay. PRF treatment has short hospital stay, less injury and strong repeatability.

## Introduction

Herpes zoster (HZ) is an infectious disease caused by varicella-Zoster virus (VZV) invading the human body and latent in the neurons of sensory ganglia such as cranial and dorsal root ganglia. When the human immune system is reduced, the latent virus is reactivated., and the accompanying pain is called herpes zoster neuralgia (1–4). Postherpetic neuralgia (PHN) is the most common complication of herpes zoster (5–7). The clinical manifestations are erythema and clustered blisters with banded distribution along one side of the peripheral nerve accompanied by periodic or persistent needle-like, knife-like and burning pain, which generally lasts for several months to several years, Pain is common in elderly patients with chronic diseases such as high blood pressure, diabetes and immune dysfunction, and most elderly patients tend to neglect the treatment of herpes zoster neuralgia after the lesion is cured, thus missing the treatment window period, and eventually leading to the occurrence of PHN, which has a serious impact on the quality of life of patients and increases the medical burden (8–10).

Although there are many treatments for herpetic neuralgia, PHN still occurs in 9%–34% of HZ patients (11, 12). However, there are limited clinical therapies that can significantly reduce the incidence of PHN with minor complications. Long-term oral drug therapy not only reduces the efficacy but also increases the toxic side effects of drugs (13–15). In order to alleviate the pain of patients with herpes zoster, clinicians have been working hard to study the minimally invasive treatment of herpes zoster neuralgia, such as nerve root block, epidural block, pulsed radiofrequency（PRF）and spinal cord electrical stimulation(SCS) (16–18).Minimally invasive methods can relieve pain, reduce peripheral and central sensitization, and prevent further deterioration of pain (19). Nerve block has a certain analgesic effect and can be treated in the outpatient department. However, the single treatment effect of nerve block is short and it is often used multiple times. Steroid drugs have no benefit on the recovery of limb PHN and motor nerve function (20).SCS is effective in the treatment of early herpes zoster neuralgia and can effectively reduce the incidence of PHN (21–24). However, due to the implantation of electrodes into the spinal canal, there is a risk of infection and electrode displacement or even fracture. In addition, patients need to pay tens of thousands of yuan of expensive out-of-pocket medical expenses, which limits its clinical application (25, 26). At present, continuous epidural block and pulsed radiofrequency are the two most commonly used methods in clinical practice. However, which method has better efficacy, fewer surgical complications and higher efficiency in preventing PHN has not yet been clarified. Therefore, the aim of this study is to compare the clinical efficacy of continuous epidural block and PRF in the treatment of acute herpes zoster neuralgia, and to explore the differences in their preventive effects on post-herpetic neuralgia.

## Methods

### Study design and ethical approval

In this study, medical records of patients who received continuous epidural block or pulsed radiofrequency therapy for acute stage herpetic neuralgia were selected from November 1, 2021, to May 23, 2022. The study protocol received approval from the Institutional Review Board of the First Hospital of Jiaxing (Approval No.: LS2021-KY-343) and was prospectively registered with the Chinese Clinical Trial Registry (Registration ID: ChiCTR2200064664). All procedures adhered to the ethical principles of the Declaration of Helsinki and Good Clinical Practice guidelines. Informed consent for this study was obtained from all participants themselves. All participants provided written informed consent and were explicitly aware of the risks and potential complications of treatment before participation in the study.

### Inclusion and exclusion criteria

The inclusion criteria were as follows: 1) met the diagnostic criteria for herpes zoster with a disease duration of <30 days; 2) herpetic neuralgia patients with cervical, thoracic or lumbar ganglion involvement; 3) visual analog scale (VAS) score > 3 points. The exclusion criteria were as follows: 1) patients with infection or tumor at the puncture site; 2) patients with severe cardiovascular and cerebrovascular diseases, liver and kidney dysfunction, or abnormal coagulation function diabetes with poor blood sugar control; 3) patients who have been using immunosuppressants for a long time, who have experienced systemic failure, and who suffer from mental disorders and are unable to cooperate with surgery; 4) patients who were lost to follow-up within 6 months after the surgery. The criteria for disqualification were as follows: 1) Lost follow-up subjects. 2) Patients who refused to undergo follow-up after surgery. 3) Patients for whom the surgical treatment was ineffective and who underwent radiofrequency cutting of the posterior root of the spinal nerve.

### Randomization and blinding

A total of 90 patients were enrolled and randomly assigned to 2 groups by computer-generated random allocation sequence. Patients who received continuous epidural block were divided into the CEB group and patients who received pulsed radiofrequency were placed in the PRF group. The two groups of patients received corresponding treatment and follow-up at different time points. The actual blinding design used in the study was single-blind-assessor-blinded. Since PRF and CEB need to be performed under CT guidance, the operator must be aware of the grouping. Patients could not be blinded because of the need to undergo a puncture procedure and because the PRF group would experience transient electrical stimulation or burning. All follow-up assessments of VAS scores and PSQI scores were performed independently by a full-time research physician who was unaware of the group assignments. This assessor was not involved in treatment or had access to treatment notes in medical records, and patients were explicitly instructed “not to disclose your treatment” before the evaluation.

### Surgical procedure

All patients were treated with oral analgesic drugs prior to surgery, and their pain remained moderate to severe (VAS Score > 3 points). All operations were performed by the same senior doctor in the pain department of our hospital.

Pulse radiofrequency: The vital signs of the patient were monitored. The segment with the most severe pain was the center, the upper and lower segments were expanded by 1 segment, and dorsal root ganglion PRF treatment was performed at 3 segments. A puncture path was designed under the guidance of CT. Lidocaine was administered for local anesthesia, puncture was performed by slowly advancing the radio-frequency trocar (20 G, 150 mm in length and 10 mm in the active end) under CT. Finally, when the needle tip was located in the ventral upper quadrant of the intervertebral foramen close to the dorsal root ganglion, root pain could occur when the puncture point reached the pain distribution area, and the three-dimensional reconstruction map clearly indicated that the puncture point was located at the target location. Then the radiofrequency instrument was connected, and sensory and exercise tests are completed. After the location of the puncture needle was confirmed, the temperature, time, pulse width and frequency of the standard voltage PRF group were set as 42°C, 300 s, 20 ms, and 2 Hz, respectively. After the end of the radiofrequency pulse, the puncture needle was drawn back without blood, gas or liquid. One hundred milligrams of 2% lidocaine hydrochloride, 1 mg of mecobalamin injection, and 1 mg of betamethasone sodium phosphate diluted with 0.9% normal saline were added to an 18 ml mixture, and 3 ml of treatment solution was injected into each segment. In all patients, the needle was extracted, and the puncture site was compressed after PRF treatment. After 30 min of observation, the patient was sent back to the ward after the vital signs were stable. After discharge, the patient continued to receive paravertebral nerve block treatment in the pain department outpatient clinic, with an interval of 3 days each time, and a total of 2 times of outpatient treatment: paravertebral puncture was performed in the corresponding segment of the patient's pain to confirm that there was no blood, gas, liquid, etc. After the puncture needle was withdrawn, 2% lidocaine hydrochloride 100 mg and mecobalamin injection 1 mg were injected, 5 ml of treatment solution was injected into each segment to complete the block.

Continuous epidural block: The patient was placed in the lateral decubitus position and abstained from food and drink for 8 h before surgery. Blood pressure (BP), heart rate (HR), electrocardiogram (ECG), and peripheral oxygen saturation (SpO2) were routinely monitored after entering the room, and peripheral venous access was opened. Epidural puncture catheterization was performed at the appropriate level under CT guidance to confirm the location of the catheter tip (Figures 4, 5). Then, 100 mg of 2% lidocaine hydrochloride, 1 mg of mecobalamin injection, 4 mg of betamethasone sodium phosphate and 0.9% normal saline were diluted to 15 ml. The injection concentration and rate of administration were adjusted according to the degree of pain relief or side effects. Two analgesic pumps were used for each patient. The formula was 0.08% ropivacaine +500 µg Micobor + normal saline diluted to 275 ml. Analgesic pump settings were as follows: maintenance volume 4 ml/h, additional volume 2 ml/time, locking time: 30 min. There was a postoperative interval of 2 days to perform blood routine, erythrocyte deposit, anterior calcitonin, and CRP examinations, and for daily dressing change at the puncture site. If signs of infection were found, the epidural catheter was immediately removed. Both groups were given 0.05 g of oral tramadol hydrochloride immediately if pain broke out during treatment.

### Data collection and follow-up

The following data were collected and analyzed: sex; age; affected side; course of disease; segment; length of hospital stay; and accompanying diseases, including hypertension and diabetes. Visual analog scores (VAS), Pittsburgh Sleep Quality Index (PSQI), dosage of gabapentin capsules and Tramadol hydrochloride sustained release tablets before surgery and 1 day, 1 week, 1 month, 3 months and 6 months after surgery. Blood galectin-3 and interleukin-6 levels before treatment and at 1, 2 and 4 weeks after treatment. The incidence of PHN, postoperative complications and length of stay were recorded in the two groups.

### Specimen collection

Specimens were collected by the same nurse with intermediate title. After collection, specimens were placed in a 4°C refrigerator and centrifuged by the subjects within 2 h. Fasting peripheral blood was collected from all subjects before treatment, 1 week after treatment, 2 weeks after treatment and 4 weeks after treatment at various time points in the morning, and all patients were kept in a quiet environment for 30 min before blood drawing, from 8:00–10 am: 4 ml peripheral venous blood was extracted and stored in an EDTA tube, mixed for 5–10 min, centrifuged for 10 min (3,000 RPM), and 2 ml of supernatant (plasma) was taken and stored in a −80°C refrigerator. Serum galectin-3 and IL-6 levels were determined by enzyme-linked immunosorbent assay (ELISA).

## Statistical analysis

### Sample size

Based on the results of the pre-experiment, the effective rates of continuous epidural block and pulsed radiofrequency therapy for acute herpes zoster neuralgia (with no occurrence of PHN as the criterion for effectiveness) were 90.0% and 70.0% respectively. That is, the recurrence rates were 10% and 30% respectively. By substituting these values into the formula, the minimum sample size was approximately 79 cases. Considering a 15% dropout rate, this study should include at least 90 patients in the research (*α* = 0.05, *β* = 0.1).

### Data analysis

SPSS 26.0 (IBM, Chicago, USA) software was used for statistical analysis. The Shapiro–Wilk test was performed to determine if the measurements were normally distributed. Data with normal distribution were expressed as mean ± standard deviation, and data with skewed distribution were expressed as median (quartile). The Mann–Whitney U test or independent sample *t* test was used for continuous variables, categorical variables were expressed as percentages, and the chi-square test or Fisher's exact test was used for categorical variables. The data of this study were analyzed using intention-to-treat methodology. *P* < 0.05 was considered statistically significant.

## Results

### Characteristics of the patients

**Figure 1:**
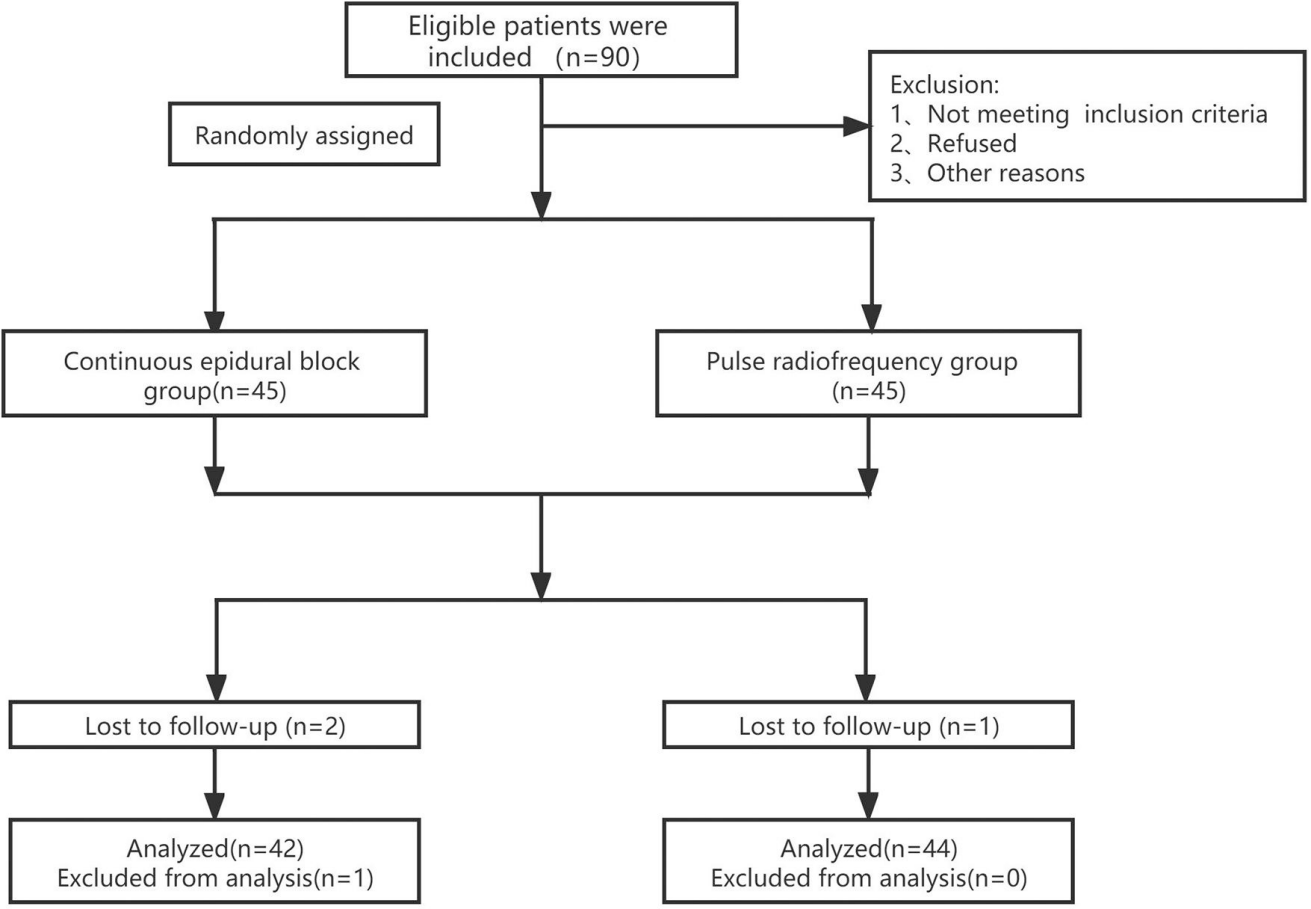
Flow chart of follow-up procedure.

A total of 90 patients were treated. Forty-five patients underwent PRF, and 45 patients underwent continuous epidural block. One case was lost to follow-up in the PRF group. In the continuous epidural group, 2 patients were lost to follow-up, and 1 patient developed puncture site infection. After excluding these patients, 86 patients were analyzed, including 44 patients in the PRF group and 42 patients in the continuous epidural block group (Figure 1). There was no significant difference in sex, age, affected side, course of disease, stage, concomitant disease, sleep quality or type of analgesic drugs between the two groups. The VAS scores before treatment were 5.53 ± 0.71 and 5.24 ± 0.76, respectively, and there was no significant difference between the two groups (*P* = 0.165, Table 1).

**Table 1:** Patients characteristics.

Variable	PRF group (*n* = 44)	CEB group (*n* = 42)	*P-Value*
Gender, *n*, (male/female)	(16/28)	(16/26)	0.849
Age (yrs), mean ± SD	63.31 ± 11.57	64.74 ± 11.07	0.270
Affected side (left/right)	(22/22)	(18/24)	0.445
Underlying disease			
Diabetes, *n* (%)	4 (9.0)	8 (19.0)	0.171
Hypertension, *n* (%)	19 (43.1)	13 (30.9)	
Diabetes + Hypertension *n*, (%)	4 (9.0)	3 (7.1)	
None n, (%)	25 (56.8)	24 (57.1)	
Course of disease (day), mean ± SD	17.14 ± 9.95	17.10 ± 9.09	0.789
Involved dermatome, n			
Cervical, *n* (%)	12 (26.2)	7 (16.7)	0.445
Thoracic, *n* (%)	27 (61.3)	28 (66.6)	
Lumbosacral, *n* (%)	5 (11.3)	7 (16.7)	
VAS score pre-operation, mean ± SD	5.53 ± 0.71	5.24 ± 0.76	0.165
PSQI score pre-operation, mean ± SD	13.68 ± 1.40	13.26 ± 1.20	0.075
Doses of medication before the procedure			
Gabapentin, (mg)	437.50 ± 173.97	442.86 ± 212.01	0.861
Tramadol, (mg)	134.09 ± 83.37	123.81 ± 75.44	0.481

### Comparison of hospitalization periods

**Figure 2:**
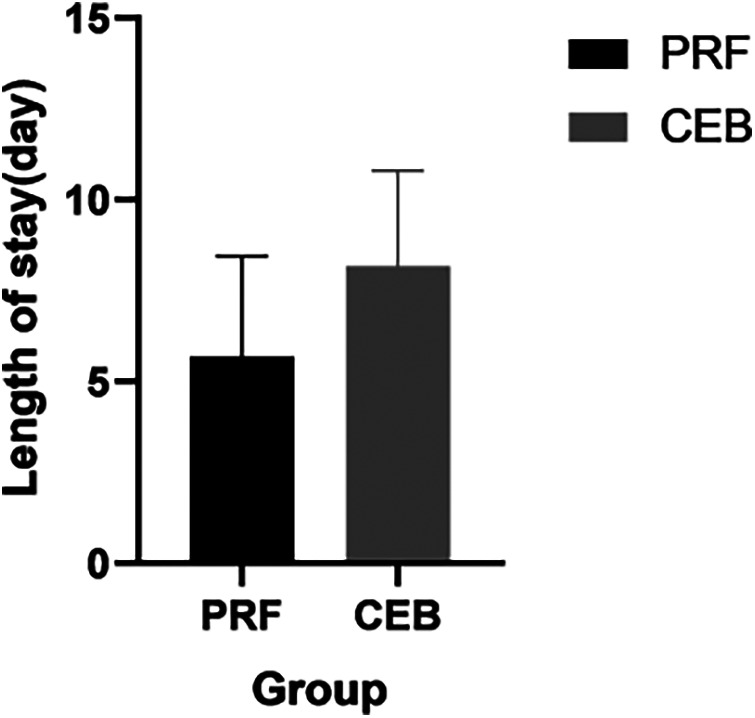
Length of hospital stay during treatment in both groups. Grey and black are the hospitalization days of CEB group and PEF group, respectively. The difference between the two groups was statistically significant (*P* = 0.000).

The length of hospital stay in the CEB group was longer than that in the PRF group (8.29 ± 2.64 and 5.26 ± 2.81, respectively), and the difference between the two groups was statistically significant (*P* = 0.000, Figure 2).

### VAS score

**Figure 3:**
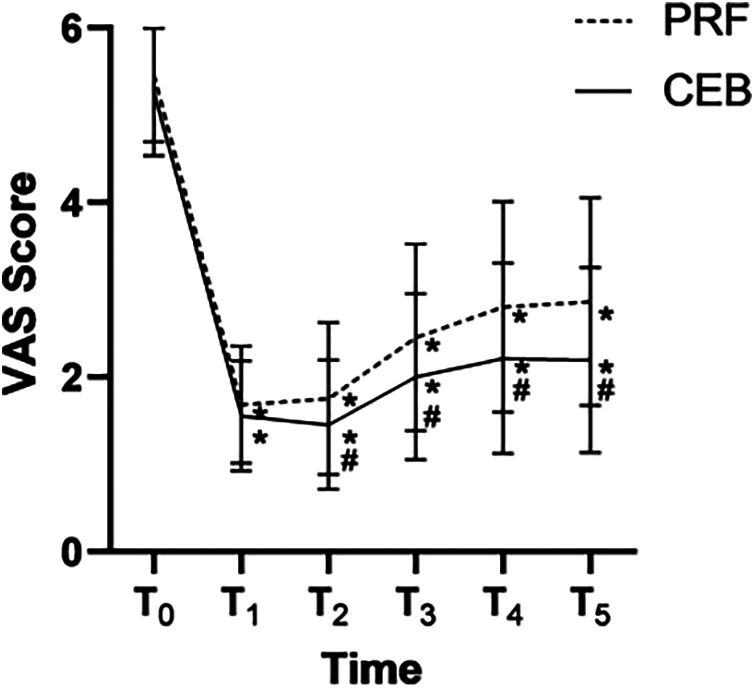
Changes in VAS after treatment. Over time, VAS decreased significantly in both groups. Compared with PRF group, VAS scores in CEB group were significantly lower at 1 week, 1 month, 3 months, and 6 months after treatment (*P* = 0.043, 0.016, 0.007, 0.001). The dotted line and the solid line represent changes in VAS in group PRF and Group CEB, respectively. *: Compared with preoperative VAS, *P* < 0.05. #: Compared with PRF group, *P* < 0.05.

Over time, VAS decreased significantly in both groups. Compared with the PRF group, VAS scores in the CEB group were significantly lower at 1 week, 1 month, 3 months and 6 months after treatment (*P* = 0.043, 0.016, 0.007, 0.001, Figure 3).

### Occurrences of PHN

**Figure 4:**
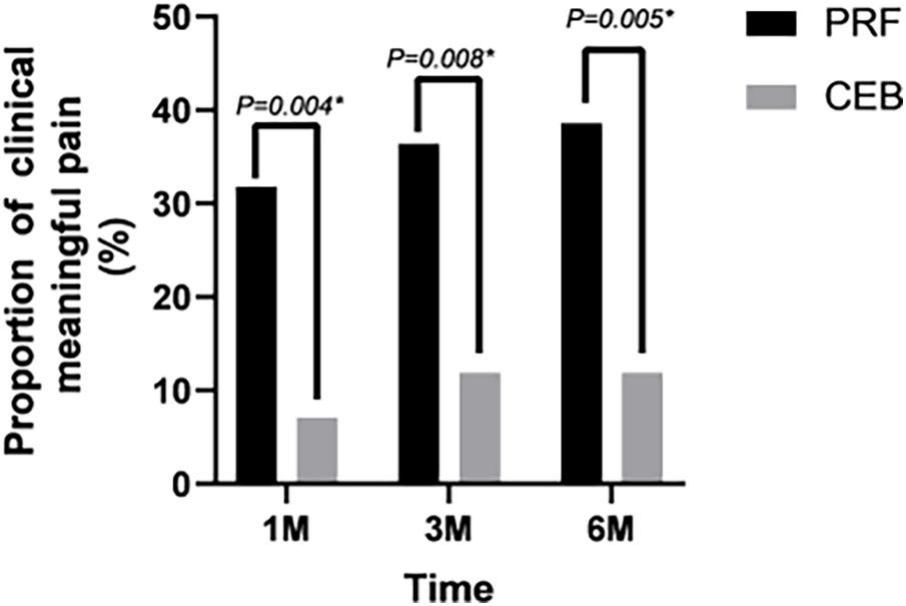
Proportion of clinically significant PHN in 2 groups 1, 3 and 6 months after treatment. Gray and black are the proportions of clinically significant PHN in CEB and PRF groups, respectively. The incidence of PHN was significantly lower in CEB group than in PRF group at 1, 3 and 6 months after treatment (*P* = 0.004, 0.008, 0.005).

The incidence of PHN was significantly lower in the CEB group than in the PRF group at 1, 3 and 6 months after treatment (*P* = 0.004, 0.008, 0.005, Figure 4).

### PSQI score

**Figure 5:**
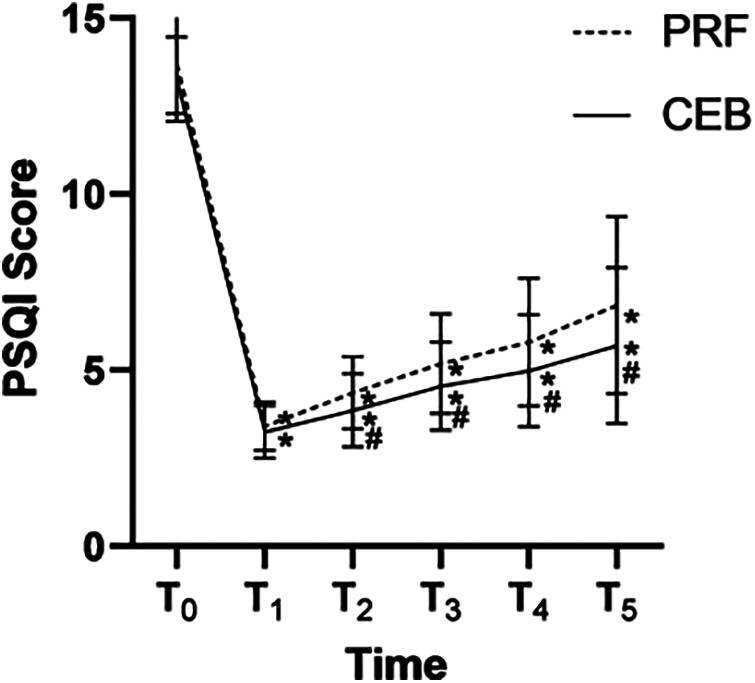
Changes in PSQI after treatment.PSQI decreased significantly in both groups. The dotted line and the solid line represent changes in PSQI in PRF and CEB groups, respectively. Compared with PRF group, PSQI scores in CEB group were significantly decreased at 1 week, 1 month, 3 months and 6 months after treatment. *: Compared with preoperative PSQI, *P* < 0.05. #: Compared with PRF group, *P* < 0.05.

The PSQI decreased significantly in both groups. Compared with the PRF group, the CEB group had significantly decreased PSQI scores at 1 week, 1 month, 3 months and 6 months after treatment (*P* < 0.05, Figure 5).

### Serum IL-6 and Gal-3 levels

**Figure 6:**
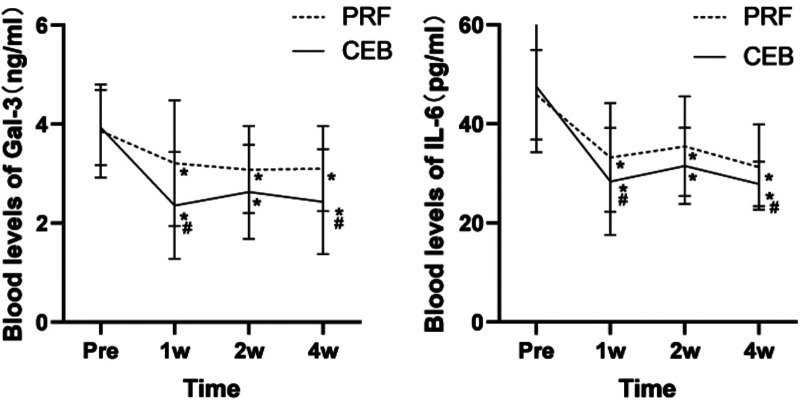
Changes in blood Gal-3 and IL-6 levels at 1, 2 and 4 weeks after treatment in both groups. The dotted line and the solid line represent changes in blood Gal-3 levels and IL-6 levels in PRF and CEB groups, respectively. *: Compared with preoperative blood Gal-3 levels and IL-6 levels, *P* < 0.05. #: Compared with PRF group, *P* < 0.05.

Compared with before treatment, the blood levels of Gal-3 and IL-6 in the 2 groups decreased at 1, 2 and 4 weeks after treatment, with statistical significance (*P* < 0.05). Compared with the PRF group, blood Gal-3 and IL-6 levels in the CEB group decreased after 1 and 4 weeks of treatment, and the difference was statistically significant (*P* < 0.05, Figure 6).

### Comparison of oral medication dosages

**Figure 7:**
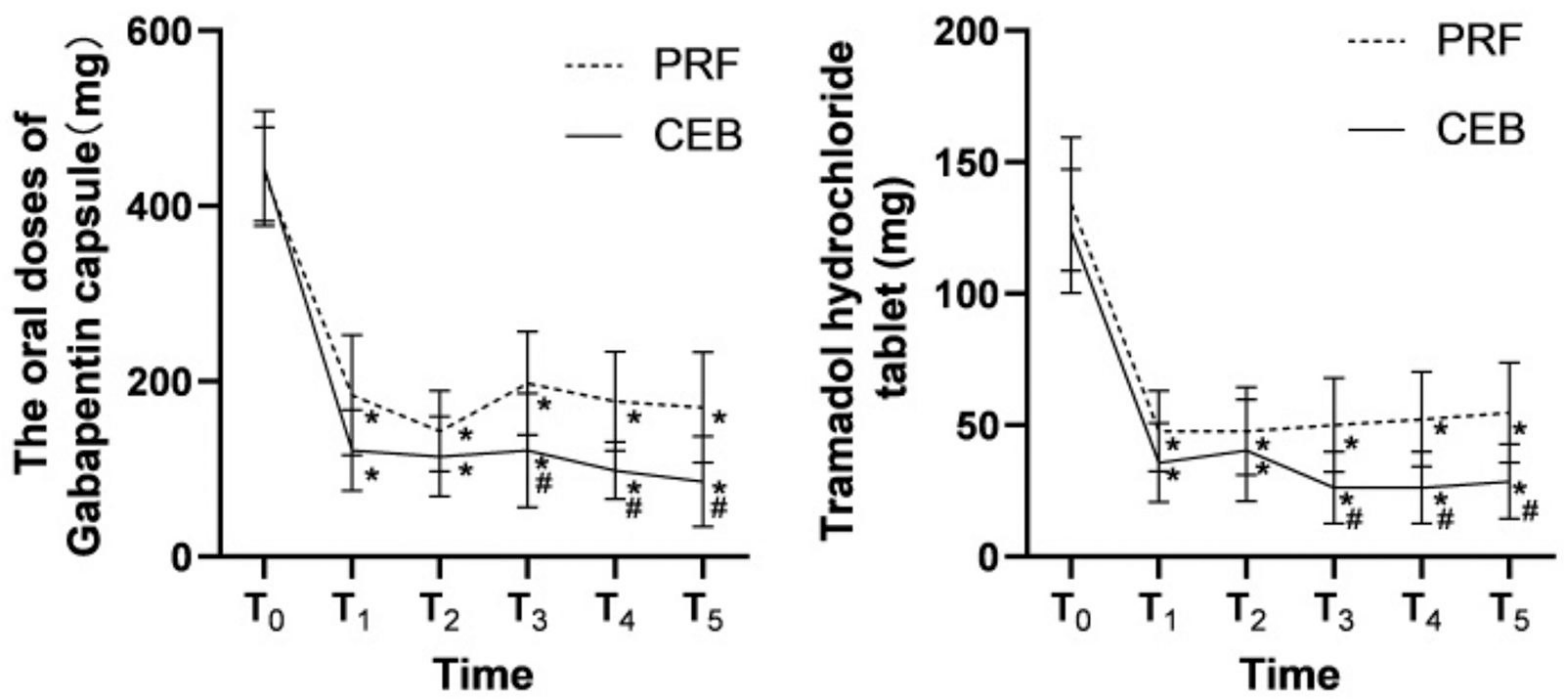
Dose changes of gabapentin capsules and tramadol hydrochloride delayed tablets after treatment. The dotted line and the solid line represent dose changes in the PRF and CEB groups, respectively. *: Compared with preoperative drug dose, *P* < 0.05. #: Compared with PRF group, *P* < 0.05.

The doses of gabapentin capsules and tramadol hydrochloride delayed release tablets decreased in both groups at 1 day, 1 week, 1 month, 3 months and 6 months after treatment. Compared with the PRF group, the oral dose of gabapentin capsules in the CEB group was decreased at 1 month, 3 months and 6 months after treatment, and the difference was statistically significant (*P* < 0.05); the oral dose of tramadol hydrochloride delayed release tablets in the CEB group was decreased at 1 month, 3 months and 6 months after treatment, and the difference was statistically significant (*P* < 0.05, Figure 7).

### The occurrence of complications

One patient in the CEB group had fever reaction 1 day after treatment, with the highest temperature of 38.4°C, redness and swelling at the puncture site, no pus was seen, and tenderness (++). Emergency blood routine test (white blood cell 12.93 × 109/L), CRP (139.7 mg/L), procalcitonin (0.23 ng/ml), and erythrocyte sedimentation rate (9 mm/h) were immediately performed. After discussion in the department, local skin infection was considered, and in order to prevent the infection focus from spreading to the spinal canal, the decision was made to immediately remove the catheter and start anti-infective treatment with oral gabapentin capsules and tramadol hydrochloride tablets. The other patients had no complications such as fever, chills, fatigue, nausea and vomiting, myalgia, and puncture injury after treatment.

## Discussion

Herpes zoster neuralgia is a type of neuropathic pain that occurs spontaneously, accompanied by an abnormal response to pain and paresthesia in the affected area. After herpes zoster infection, the virus damages sensory neurons, resulting in changes in the composition, distribution, and function of their transmembrane ion channels. Damaged sensory neurons fire, and these signals travel to the spinal cord, causing spontaneous pain (27).Neuropathic pain caused by shingles brings great physical and mental torture to patients (28). It not only reduces the living standard and work efficiency of patients but also increases the economic burden. Morbidity and hospitalization rates are also gradually increasing.Herpes zoster neuralgia is most common in elderly individuals, and age is one of the most important risk factors for the onset of herpes zoster, which may be related to tissue degeneration and decreased cellular immune function in elderly individuals, resulting in low repair ability after nerve injury and increased repair time (29).In addition, decreased immune function in elderly individuals leads to poor antiviral ability, so it should be treated in time before transforming into postherpetic neuralgia. Interventions that reduce painful irritation and inflammation during the acute phase of herpes zoster may reduce central sensitization and substantially reduce the incidence of chronic pain development. Prompt intervention leads to faster resolution of inflammation and reduced pain stimulation, which is the reason for prevention of PHN (30, 31). The most common preventative measure is drug therapy, but it is not fully effective in preventing PHN (32). The two most commonly used methods are continuous epidural block and pulsed radiofrequency. This study was to compare the efficacy of continuous epidural block and pulsed radiofrequency in the treatment of acute herpes zoster neuralgia and the prevention of PHN.

Continuous epidural block can not only effectively eliminate pain, reduce the amount of oral drugs, accelerate the healing of herpes scabs and shorten the course of treatment but also greatly improve the effective rate of the treatment of herpes zoster neuralgia. After successful epidural puncture and catheterization, local anesthetics are injected, which can reduce nerve excitability, repair the damage of herpes virus to nerve roots, and block the conduction of sensory nerves,and make an effective analgesic effect (33, 34). Currently, it is believed that the possible mechanism of epidural block in the treatment of herpetic neuralgia is to block the virus from entering the nervous system in the form of retroaxial axons, inhibit the pain signal transmission from free sensory nerve endings, block the sympathetic nerve, and improve the nutrient blood vessels innervating the spinal nerve to relieve and block the vicious pain cycle (35).However, this treatment method still has some shortcomings, such as the risk of infection caused by continuous epidural catheterization. In this study, one patient developed puncture site infection, possibly due to the patient's advanced age, decreased immunity and resistance, and bacterial invasion into the skin. We immediately performed emergency blood routine, CRP, procalcitonin and erythrocyte deposition examinations and found that the patient's inflammatory indicators increased, and we immediately removed the catheter. This prevented the lesion from spreading to the spinal canal and causing serious consequence. In this study, there was a patient who underwent continuous epidural block for the acute stage of lumbar and back herpetic neuralgia, but the postoperative pain was still unbearable, and the patient was given radiofrequency amputation of the posterior spinal nerve root, which effectively relieved the patient's touch-induced pain.

Pulsed radio frequency (PRF) limits heating to less than 42˚C,by using a pulsed current, with little risk of heat damage or nerve damage. At present, PRF has been applied to many acute and chronic pain diseases, and has achieved certain therapeutic effect. Regarding pulsed radiofrequency, studies have shown that it can enhance the descending pain inhibition pathway of norepinephrinergic and serotonergic neurons and inhibit excitatory and injury-inducing C fibers (36). The activity of microglia in the dorsal horn of the spinal cord decreased after the application of pulsed radiofrequency to the dorsal root ganglia. Since microglia are involved in chronic neuropathic pain by releasing various cytokines and chemokines associated with pain signals, the authors suggest that downregulation of microglia may alleviate chronic neuropathic pain (37). PRF is a minimally invasive treatment method with accurate positioning, few complications, strong selectivity and a high safety factor (38). In this study, patients in the PRF group were given rescue paravertebral nerve block in the outpatient clinic shortly after discharge, and this measure did not affect the efficacy results observed in the PRF group. Because a single paravertebral block uses a local anesthetic such as lidocaine, its direct analgesic effect usually lasts only a few hours. However, our primary outcome measures were at 1, 3, and 6 months after discharge. The two short blocks did not explain the reduction in pain relief and incidence of PHN over a period of up to half a year. Second, both blocks were performed after hospital discharge, when the worst pain of the acute phase had passed. The existing literature shows that there is a lack of evidence for the long-term efficacy of intermittent nerve block in the prevention of subacute PHN, while the neuromodulatory effects of PRF have a more durable biological basis for the efficacy of herpes zoster patients (regulation of c fibers and reduction of pain production substance release). A large number of clinical studies have been reported on the treatment of acute herpetic neuralgia or PHN with dorsal root ganglion pulse radiofrequency, which has also confirmed the effectiveness and safety of dorsal root ganglion pulse radiofrequency (39–41). In this study, 17 patients received PRF in the treatment of acute herpes zoster neuralgia, but the effect was not good. We gave the patients a gabapentin capsule and tramadol hydrochloride dose, which effectively relieved the pain of the patients.

Studies have shown that blood levels of galectin-3 and IL-6 are positively correlated with the severity of neuropathic pain in patients (42). Previous studies of our research group found that plasma levels of Gal-3 and IL-6 in patients with herpes zoster were correlated with early herpes zoster neuralgia and could predict the occurrence of PHN (27, 39). In this study, the levels of Gal-3 and IL-6 in blood of patients with acute herpes zoster neuralgia treated by continuous epidural block and pulsed radiofrequency decreased 1, 2, and 4 weeks after treatment compared with before treatment, indicating that patients' pain was relieved and their immunity improved. Continuous epidural block decreased more significantly than pulsed radio frequency at 1 week and 4 weeks after treatment, which may be because continuous epidural block could block the virus entering the nervous system and continuously inhibit the transmission of pain signals, thereby reducing inflammatory pain and reducing the levels of Gal-3 and IL-6 in peripheral blood.

In addition, patients with acute herpetic neuralgia were treated with continuous epidural block for longer hospital stays. PRF treatment has a shorter hospital stay and better repeatability (27). That is one of the things that we think about when we choose treatment.

### Limitations

The main limitation of this study is that all cases were from the same center and the number of patients was relatively small. Multi-center studies with larger samples are needed in the future.

## Conclusions

The results of this study suggest that both pulsed radiofrequency therapy and continuous epidural block therapy can effectively relieve the pain of patients with acute herpes zoster neuralgia, and the pain relief degree of continuous epidural block is more significant than that of pulsed radiofrequency group, and continuous epidural block is more effective in preventing PHN. The possible reason is that continuous epidural blocks can continuously block pain signaling and reduce allergic reactions to pain. However, pulsed radiofrequency has fewer complications than continuous epidural blocks, which carry a risk of intraspinal infection.

## Data Availability

The original contributions presented in the study are included in the article/Supplementary Material, further inquiries can be directed to the corresponding author/s.
